# Control charts using half-normal and half-exponential power distributions using repetitive sampling

**DOI:** 10.1038/s41598-023-50137-w

**Published:** 2024-01-02

**Authors:** Muhammad Naveed, Muhammad Azam, Nasrullah Khan, Muhammad Aslam, Muhammad Saleem, Muhammad Saeed

**Affiliations:** 1 Department of Statistics, National College of Business Administration and Economics, Lahore, 54660 Pakistan; 2 Department of Statistics, Govt Graduate College (B) Gulberg, Lahore, Pakistan; 3 Department of Statistics and Computer Science, University of Veterinary and Animal Sciences, Lahore, 54000 Pakistan; 4 College of Statistical Science, University of the Punjab, Lahore, Pakistan; 5 Department of Statistics, Faculty of Science, King Abdulaziz University, 21551 Jeddah, Saudi Arabia; 6 Department of Industrial Engineering, Faculty of Engineering-Rabigh, King Abdulaziz University, 21589 Jeddah, Saudi Arabia; 7 Department of Statistics, Minhaj University, Lahore, 54770 Pakistan

**Keywords:** Engineering, Mathematics and computing

## Abstract

This manuscript presents the development of an attribute control chart (ACC) designed to monitor the number of defective items in manufacturing processes. The charts are specifically tailored using time-truncated life test (TTLT) for two lifetime data distributions: the half-normal distribution (HND) and the half-exponential power distribution (HEPD) under a repetitive sampling scheme (RSS). To assess the effectiveness of the proposed control charts, both in-control (IC) and out-of-control (OOC) scenarios are considered by deriving the average run length (ARL). Various factors, including sample sizes, control coefficients, and truncated constants for shifted phases, are taken into account to evaluate the performance of the charts in terms of ARL. The behavior of ARLs is analyzed in the shifted process by introducing shifts in its parameters. The superiority of the HEPD-based chart is highlighted by comparing it with both the HND-based ACC and the ACC based on the Exponential distribution (ED) under TTLT using RSS. The results showcase the superior performance of the proposed HEPD-based chart, indicated by smaller ARL values. Additionally, the benefits of another proposed ACC using HND are compared with the ED-based ACC under RSS, further confirming the effectiveness of the HND-based approach through smaller ARLs Finally, the proposed control charts are evaluated through simulation testing and real-life implementation, emphasizing their practical applicability in real-world manufacturing settings.

## Introduction

Control charts play a fundamental role in ensuring and improving the quality of manufacturing items. These charts consist of upper and lower limits, within which manufacturing operations should ideally fall to be considered under control. However, numerous factors, such as machine errors, temperature fluctuations, or untrained staff, can disrupt the process, leading to shifts in target mean, variance, or both. Control charts are employed to detect such variations in ongoing processes. By promptly identifying these changes, engineers can take remedial measures to adjust or eliminate the causes of variations, ultimately enhancing the company's standards and profitability.

Control charts are tailored to suit the specific nature of the quality characteristic of a product, which can be discrete, attribute-based, or continuous. Attribute control charts (ACCs) are utilized for categorical data, such as the count of non-conforming items, while variable control charts are employed for continuous data. The attribute control chart (ACC) offers an advantage over the variable control chart (CC) by providing a faster analysis of outcomes while reducing both cost and time requirements. This advantage stems from the fact that ACCs only require classifying units as either good or defective, eliminating the need for precise measurements. Due to these benefits, ACCs have been extensively studied by numerous researchers. The construction of these charts typically assumes that the quality features follow a normal distribution. However, there are instances where the quality traits are associated with the lifespan of manufacturing items, which may exhibit non-normal distributions.

To address such scenarios, researchers have proposed ACCs that incorporate life tests, particularly time truncated life test (TTLT), as a cost-effective and time-saving experimental approach. A TTLT is a crucial method for evaluating a product's reliability or durability within a specific timeframe. It's especially valuable when testing until all units fail becomes impractical or expensive. Instead, this approach concludes at a predetermined time, focusing on meticulously counting and statistically examining failures during this period to glean insights into the product's reliability. This process involves key steps. Initially, researchers determine the test duration, considering factors like the expected lifespan of the product or the time needed to gather relevant failure data. Following this, they meticulously record and analyze the failures occurring within the defined timeframe. Subsequently, statistical methods are employed to estimate failure rates, probabilities, or reliability metrics based on these observed failures within the truncated timeframe. This comprehensive analysis aids in understanding the product's reliability within the specified duration, offering crucial insights for assessment. The concept of an ACC based on the Weibull distribution (WD) for life data, utilizing time TTLT, was first introduced by Aslam and Jun^1^. The researchers constructed various tables to predefine ARLs as a means of assessing the performance of the ACC. To further evaluate the feasibility and effectiveness of their approach, simulation study and practical example were also conducted and discussed. Similarly, Arif Osama and Aslam^2^ applied TTLT to life time data following the exponentiated Weibull distribution. Furthermore, Rao^3^ developed an ACC using TTLT for the exponentiated half logistic distribution. In a separate study, Aslam et al.^4,5^ employed TTLT in the Pareto distribution of the second kind for the development of the ACC chart. The researchers made the assumption that the lifetime of the product followed this specific distribution, with either a known or unknown shape parameter.

Further research on the application of TTLT can be observed in the work of Al-Marshadi et al.^6^. The authors introduced an ACC utilizing a neutrosophic WD to enhance the monitoring of the process with increased efficiency. The performance of this ACC was evaluated by analyzing the run length characteristic, focusing on the detection of shifts in uncertainty and the indeterminacy effect. Zaka et al.^7^ focused on developing and evaluating the ACC using two different life data distributions, transmuted power function distribution (TPFD) and survival weighted Power function distribution (SWPFD), under a TTLT. Through comparative and simulation studies, they concluded that the CCs based on SWPFD were more effective in monitoring non-conforming items compared to those based on TPFD.

In a recent study, Naveed et al.^8^ introduced an ACC that utilized two life data distributions, namely the half normal distribution and the half exponential power distribution, in conjunction with TTLT. The aim of their research was to assess the performance of the proposed ACC. To evaluate its effectiveness, the out-of-control ARLs were calculated. This measure helps determine how quickly the ACC can detect and signal out-of-control conditions in the manufacturing process. In addition to the theoretical analysis, the researchers conducted practical examples and a simulation study to further validate the proposed approach. Practical examples involved applying the ACC to real-world manufacturing scenarios, providing insights into its applicability and effectiveness in different practical settings. On the other hand, the simulation study allowed for controlled experiments, enabling the researchers to evaluate the performance of the ACC under various conditions and scenarios.

Control charts that possess the ability to promptly detect minor shifts are considered more effective compared to other control charts. To enhance the efficiency of control charts in capturing smaller variations, researchers have explored different sampling techniques. One such technique that has gained significant attention is the repetitive sampling scheme (RSS). RSS is favored for its simplicity and superior performance in detecting minor shifts in a process. By utilizing RSS, control charts become more sensitive to minor changes, providing early warnings when the process begins to exhibit any deviations from the expected behavior. The concept of RSS was introduced by Sherman^9^ and has since been widely applied in acceptance sampling plans by researchers such Aslam et al.^10–13^, Balamurali and Jun^14^, Balamurali et al.^15^ and Yen et al.^16^ further extended the application of RSS by developing control charts using the concept of RSS. The control chart using RSS used four control limits (CLs) instead of the two conventional limits. This innovative approach involves the inclusion of both inner and outer control limits, namely ${LCL}_{2}{, UCL}_{2} and {LCL}_{1} , {UCL}_{1}$. These multiple limits allow for a more nuanced identification of small and moderate fluctuations in process parameters.

In executing the RSS-based sampling CC method, certain rules are established. If the statistical value falls within the inner control limits ${LCL}_{2}{ and UCL}_{2},$ the process is deemed to be in control. On the other hand, if the computed value surpasses the outer control limits ${LCL}_{1} and {UCL}_{1}$, the process is considered uncontrolled. However, the complexity arises when the statistic value falls between ${LCL}_{1}$ and ${LCL}_{2}$ or between ${UCL}_{2}$ and ${UCL}_{1}$, In this scenario, the sampling process continues until a conclusive determination of a controlled or uncontrolled process is reached. The evaluation of this RSS-based CC is notably advantageous as it efficiently recognizes various types of process variations, contributing to a more comprehensive understanding of process control and aiding in making informed decisions about process adjustments. The authors proposed the T-chart with RSS and compared its performance in terms of average run length (ARL) with the approach presented by Santiago and Smith^17^, demonstrating the superior performance of their proposed method with lower ARL values.

After that Aslam et al.^11,12^ developed variable as well as ACC using RSS. The utilization of RSS can also be observed in studies conducted by Aslam et al.^4,18^, Azam et al.^19,20^, Lee et al.^21^ and Rao et al.^22^ introduced the idea of an attribute control chart (ACC) for the Birnbaurn-Saunders distribution under time truncated life tests (TTLT) using RSS. Additionally, Jeyadurga et al.^23^ applied the concept of RSS to life data distributions and constructed an ACC for the Pareto distribution of the second kind. Tanveer et al.^24^ focused on the development and evaluation of the ACC using resampling techniques (Repetitive sampling and multiple dependent state) for monitoring non-conforming items under the exponentiated half-logistic distribution. The derived formulas, extensive ARL tables, real-life example, and simulation study collectively highlight the effectiveness of the proposed control chart in detecting assignable causes in a timely manner. Adeoti and Rao^25^ presented an ACC that incorporates RSS under TTLT for monitoring the mean life of a product. The proposed chart exhibits improved performance compared to existing control charts, as evidenced by the ARL values. The study provides valuable insights and a practical example, highlighting the potential application and benefits of the proposed ACC in real-world manufacturing processes. Recently, Saleh et al.^26^ proposed that repetitive sampling methods can be useful, however, when monitoring with count data. More information on the use of control charts can be seen in Lee et al.^21^, Tanveer et al.^24^, Elal-Olivero et al.^27^, Naveed et al.^28^ and Rao et al.^29,30^.

Building upon prior research investigating the utilization of ACC with TTLT under RSS in manufacturing industries, our objective is to develop ACCs using two well-known life data distributions: the half-normal distribution and the half-exponential power distribution. It is worth mentioning that these particular distributions have not been thoroughly explored in existing literature pertaining to the development of control charts under TTLT using RSS. The paper is structured as follows: In Sect. 2, a concise overview of the two life data distributions is provided to establish a foundation for the subsequent sections. Section 3 delves into the proposed design of the control chart, taking into consideration the scenario where the parameters of the chart are shifted. The advantages of the presented control chart are discussed in Sect. 4, highlighting its strengths and benefits in comparison to other approaches. Section 5 presents the results of a simulation study that was conducted to assess the effectiveness and performance of the suggested control chart. In Sect. 6, a real-world application of the recommended control chart is presented, demonstrating its practical utility in a specific context. Finally, Sect. 7 contains concluding remarks, summarizing the key findings and implications of the study.

## Introduction of two life data distribution

This section provides a concise introduction about two important distributions: the Half-Normal Distribution (HND) and the Half-Exponential Power Distribution (HEPD). In Sect. 2.1, we explore the application of the HND in the realm of statistical quality control. Furthermore, in Sect. 2.2, we delve into the application of the HEPD within the field of statistical quality control. These discussions shed light on the significance and relevance of these distributions in the context of quality control practices.

### Half Normal Distribution (HND)

The Half Normal Distribution (HND) is a widely employed statistical distribution commonly used for modeling life data. It was first developed and studied by Chou and Liu^31^, who extensively examined its properties and explored its applications in the field of quality control. Notably, the HND finds particular relevance when dealing with data related to fatigue, which refers to the structural deterioration that occurs when a material is consistently subjected to stress. In such cases, the HND serves as a valuable tool for analyzing and understanding the characteristics of fatigue-related data^32^. It is worth noting that the HND is a special case of the normal distribution, showcasing its significance and applicability in various statistical contexts. The Half Normal Distribution (HND) is a special case of Normal Distribution with mean 0 and its scale parameter $\alpha$ which is limited to the domain $[ 0,\infty )$. The probability distribution of half normally distributed variable $t$ is given by

1 $$f\left(t,\alpha \right)=\frac{1}{\alpha }\sqrt{\frac{2}{\pi }}exp\left(\frac{-{t}^{2}}{2{\alpha }^{2}}\right) , t\ge 0, \alpha >0$$

here $\alpha$ is the scale parameter. The CDF denoted by $F\left(t\right)$ is given by

2 $$F\left(t\right)=erf\left(\frac{t}{\alpha \sqrt{2}}\right)$$

The $erf$ is the error function defined as

3 $$erf\left(x\right)=\frac{2}{\sqrt{\pi }}\underset{0}{\overset{x}{\int }}{\text{exp}}(-{t}^{2}) dt$$

The hazard rate function, denoted as $h\left(t\right)$, for a continuous distribution is defined as the ratio of the probability density function (PDF) to the survival function, given by

4 $$h\left(t\right)=\frac{f\left(t\right)}{1-F\left(t\right)}$$

**Figure 1:**
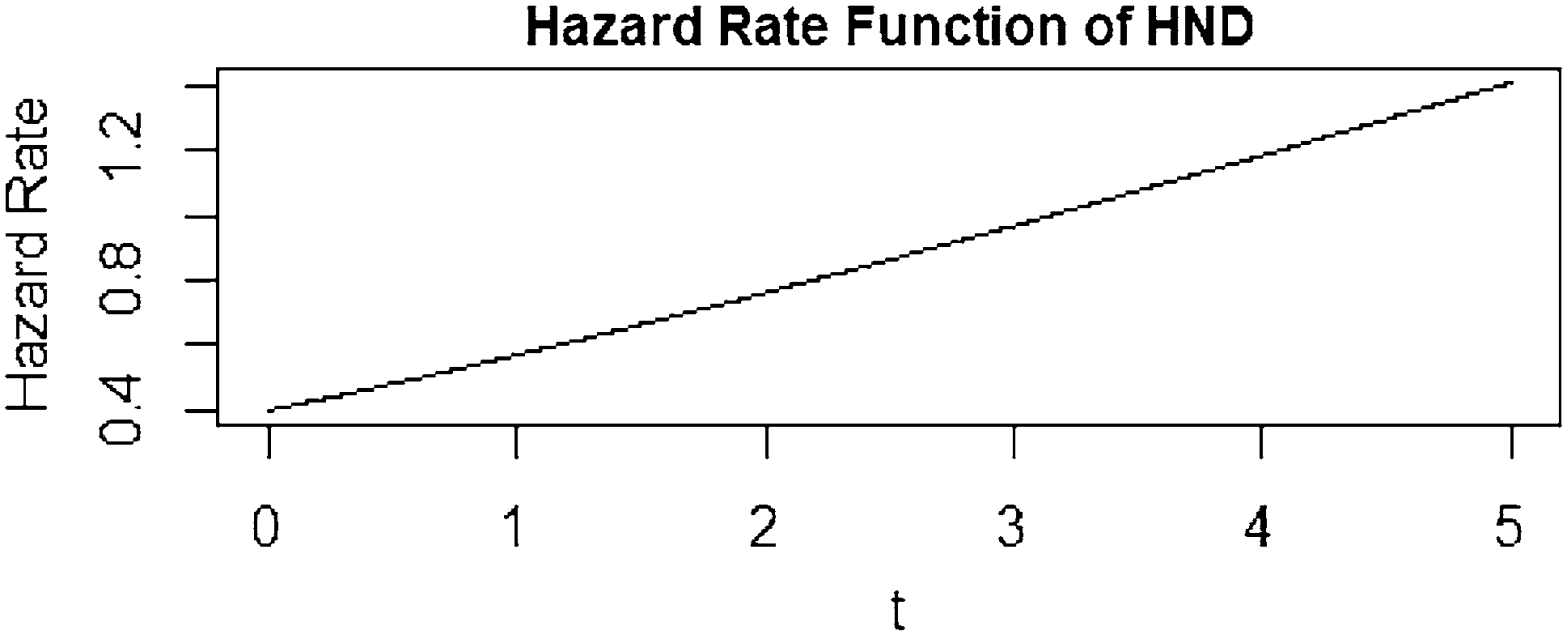
Graph of hazard rate function of HND when α = 2.

Substituting the PDF and CDF of the HND into this formula will yield the hazard rate function for the HND and the graph of hazard rate function is also shown in Fig. 1

5 $$h\left(t\right)=\frac{\frac{1}{\alpha }\sqrt{\frac{2}{\pi }}exp\left(\frac{-{t}^{2}}{2{\alpha }^{2}}\right)}{1-erf\left(\frac{t}{\alpha \sqrt{2}}\right)}$$

The positive trend in the hazard rate function graph for the half-normal distribution indicates an increasing failure rate with higher values of the random variable. This behavior portrays a growing likelihood of failure as the variable values progress, suggesting an escalating risk associated with larger values. Despite starting from zero, the hazard rate consistently rises as the variable increases, signifying a continuous elevation in the failure rate. The mean and variance of HND are given by

6 $$\mu =E\left(t\right)=\alpha \sqrt{\frac{2}{\pi }}$$

7 $$var\left(t\right)=\left(\frac{\pi -2}{\pi }\right){\alpha }^{2}$$

The versatile application of the Half Normal Distribution (HND) extends beyond quality control. It finds utility in various fields such as sports science, blowflies, physiology, stochastic frontier models, fiber buckling, and notably, reliability analysis. The significance of HND in Acceptance Sampling Plans can be observed in research studies conducted by Azam et al.^19,20^, Rao et al.^30^, Al-Omari et al.^33^ and Lu et al.^34^. These studies highlight the diverse range of applications where the HND plays a crucial role, contributing to advancements and insights in various scientific disciplines.

### Half Exponential Power Distribution (HEPD)

The Half-Exponential Power Distribution (HEPD) is a distribution that arises from truncating the Exponential Power Distribution, developed by Gui^35^. It serves as a generalized distribution that encompasses both the Half Normal Distribution (HND) and the Exponential Distribution (ED), specifically designed for non-negative variables. The HEPD finds extensive application in the fields of reliability analysis and quality control. In a study conducted by Gui^36^, a sampling plan for the HEPD utilizing the Time Truncated Life Test (TTLT) concept was developed. This plan provides guidelines for effective sampling and analyzing data from the HEPD distribution. Additionally, Gui and Xu^37^ proposed a double acceptance sampling plan specifically tailored for the HEPD using TTLT. This plan aims to further enhance the efficiency and effectiveness of the sampling process in the context of the HEPD distribution. More recently, Naveed et al.^8^ proposed the use of the HND and HEPD distributions in the development of an ACC. This further highlights the practical significance and broad applicability of the HEPD in reliability analysis and quality control applications. Overall, the HEPD is a crucial distribution that expands the range of applications for the HND and ED of non-negative variables. Its utilization in reliability analysis and quality control, as demonstrated by the aforementioned studies, underscores its practical importance in various fields. The probability distribution function (pdf) and cumulative distribution function (cdf) of HEPD are given by

8 $$f\left(t\right)=\frac{{\lambda }^{1-\frac{1}{\lambda }}}{\alpha\Gamma \left(\frac{1}{\lambda }\right)}{e}^{-\frac{{t}^{\lambda }}{\lambda {\alpha }^{\lambda }}};t\ge 0, \alpha >0, \lambda >0.$$

9 $$\frac{{F\left( t \right) = \gamma \left( {\frac{1}{\lambda },\frac{{t^{\lambda } }}{{\lambda \alpha ^{\lambda } }}} \right)}}{{\Gamma \left( {1/\lambda } \right)}}$$

**Figure 2:**
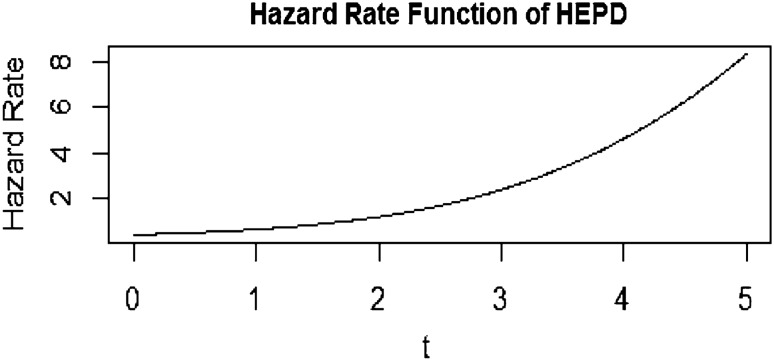
Graph of hazard rate function of HEPD when α = 2 and λ = 4.

Here, the parameter $\alpha$ represents the scale parameter, while $\lambda$ represents the shape parameter. The HEPD distribution exhibits different characteristics depending on the values of these parameters. Specifically, when the shape parameter $\lambda$ is equal to 1, the HEPD is equivalent to the Exponential Distribution (ED). Similarly, when $\lambda$ takes the value of 2, the HEPD transforms into the HND. The hazard rate function for the HEPD is given as

10 $${\text{h}}\left( {\text{t}} \right) = \frac{{\frac{{\uplambda ^{{1 - \frac{1}{\uplambda }}} }}{{\upalpha \Gamma \left( {\frac{1}{\uplambda }} \right)}}{\text{e}}^{{ - \frac{{{\text{t}}^{\uplambda } }}{{\uplambda \upalpha ^{\uplambda } }}}} }}{{1 - \frac{{\gamma \left( {\frac{1}{\uplambda },\frac{{{\text{t}}^{\uplambda } }}{{\uplambda \upalpha ^{\uplambda } }}} \right)}}{{\Gamma \left( {1/\uplambda } \right)}}}}$$

we also display the graph of hazard rate function of HEPD in Fig. 2.

The positive curve linear trend in the hazard rate function (HRF) of the half-exponential power distribution signifies a consistent and steady increase in the failure rate over time or across variable values, depicted by a curved linear pattern in the HRF graph. The mean of HEPD is as follows

11 $$\mu = E\left( t \right) = \frac{{\alpha \lambda ^{{1/\lambda }} }}{{\Gamma \left( {1/\lambda } \right)\Gamma \left( {2/\lambda } \right)}}$$

Let the average life time of the failure products for in-control (IC) process is ${\mu }_{0}$.

## Designing of proposed control charts

This section introduces two types of ACCs developed for monitoring purposes. These ACCs are specifically designed to utilize the HND and HEPD under the concept of TTLT using RSS. We begin by detailing the construction procedure for the ACC based on HND. This includes the step-by-step process involved in establishing and implementing the control chart using HND. Next, we proceed to outline the construction procedure for the ACC based on HEPD. This procedure provides a comprehensive guide on how to build and employ the control chart based on the HEPD distribution.

### Designing of the control chart using Half Normal Distribution

Here, we suppose that the failure time of manufacturing item denoted by $t$ follows the HND. The probability that an item fails before time ${t}_{0}$ is given by

12 $$P\left(t<{t}_{0}\right)=erf\left(\frac{t}{\alpha \sqrt{2} }\right)$$

replace the value of ${t}_{0}=q{\mu }_{0}$ where $q$ indicates the truncated constant for HND and the value of $\alpha$ in term of $\mu$ using Eq. (6), then Eq. (12) can be written as

$$P\left(t<{t}_{0}\right)=erf\left(\frac{q{\mu }_{0}}{\frac{\mu \sqrt{\pi }}{\sqrt{2} }\sqrt{2}}\right)$$

13 $$P\left(t<{t}_{0}\right)=erf\left(\frac{q}{\sqrt{\pi } }\frac{{\mu }_{0}}{\mu }\right)$$

The process is considered to be IC when $\mu ={\mu }_{0}$ then Eq. (13) becomes

14 $${P}_{0HND}=P\left(t<{t}_{0}\right)=erf\left(\frac{q}{\sqrt{\pi }}\right)$$

The inner and outer lower and upper control limits for proposed $np$ charting structure using HND under RSS is as follows:

The outer control limits for proposed chart are given as

15 $${LCL}_{1}={\text{max}}\left[n{P}_{0HND}-{W}_{1}\sqrt{n{P}_{0HND}\left(1-{P}_{0HND}\right)}\right]$$

16 $${UCL}_{1}=n{P}_{0HND}+{W}_{1}\sqrt{n{P}_{0HND}\left(1-{P}_{0HND}\right)}$$

The inner control limits for proposed chart are given as

17 $${LCL}_{2}={\text{max}}\left[n{P}_{0HND}-{W}_{2}\sqrt{n{P}_{0HND}\left(1-{P}_{0HND}\right)}\right]$$

18 $${UCL}_{2}=n{P}_{0HND}+{W}_{2}\sqrt{n{P}_{0HND}\left(1-{P}_{0HND}\right)}$$

Here ${W}_{1} and {W}_{2}$ are the control coefficients with ${W}_{1}$ being greater than ${W}_{2}$. These coefficients are determined based on the desired ARL for an in-control process. It is important to note the if ${W}_{1}={W}_{2}$ the proposed chart is converted to Naveed et al.^8^ based on HND.

The working procedure for the presented chart is as follows:

Step 1: Randomly select a sample of size $n$ from each subgroup and conduct the TTLT with a duration of ${t}_{0}$. Count the number of items that fail before time ${t}_{0}$ and denote it as $X$.

Step 2: Determine the control status based on the value of $X$. If $X$ falls below the outer lower control limit ${LCL}_{1}$ or exceeds the outer upper control limit ${UCL}_{1}$, the production process is considered out-of-control (OOC). If $X$ falls within the range of the inner lower control limit ${LCL}_{2}$ and the upper control limit ${UCL}_{2}$, the ongoing operation is considered in-control (IC). If $X$ falls between ${LCL}_{1}$ and ${LCL}_{2}$ or between ${UCL}_{2}$ and ${UCL}_{1}$, repeat Step 1.

It is important to note that the number of failure items $X$ for the IC process follows the Binomial Distribution (BD) with parameters $n$ and ${P}_{0HND}$ Here, ${P}_{0HND}$ represents the probability of an item fails before time ${t}_{0}.$ In practical situations, the value of ${P}_{0HND}$ is often unknown, To establish control limits that are applicable in real-world scenarios, a preliminary sample is taken from the IC process in order to estimate the value of ${P}_{0HND}.$ This preliminary sample helps in determining the appropriate control limits for subsequent monitoring and control purposes.

The inner and outer control limits are then defined as follows:

19 $${LCL}_{1}={\text{Max}}\left[0, \overline{X }-{W}_{1} \sqrt{\overline{X }\left(1-\overline{X }/n\right)}\right]$$

20 $${UCL}_{1}=\overline{X }+{W}_{1} \sqrt{\overline{X }\left(1-\overline{X }/n\right)}$$

21 $${LCL}_{2}={\text{Max}}\left[0, \overline{X }-{W}_{2} \sqrt{\overline{X }\left(1-\overline{X }/n\right)}\right]$$

22 $${UCL}_{2}=\overline{X }+{W}_{2} \sqrt{\overline{X }\left(1-\overline{X }/n\right)}$$

where $\overline{X }=\frac{\sum X}{n}$ denotes the mean failure time of items before time ${t}_{0}$ in a subgroup over a preliminary sample.The probability of declaring the running operation as out-of-control (OOC) under RSS when it is actually in control, is given as

$${P}_{out,0}^{o}= P\left(X<{LCL}_{1}|{P}_{0HND}\right)+P\left(X>{UCL}_{1}|{P}_{0HND}\right)$$

23 $${P}_{out,0}^{o}=\sum_{x=0}^{{LCL}_{1}}\left(\genfrac{}{}{0pt}{}{n}{x}\right) {\left(erf\left(\frac{q}{\sqrt{\pi } }\right)\right)}^{x}{\left(1-erf\left(\frac{h}{\sqrt{\pi } }\right)\right)}^{n-x}+\sum_{{d}_{1}={UCL}_{1}+1}^{{LCL}_{1}} \left(\genfrac{}{}{0pt}{}{n}{x}\right) {\left(erf\left(\frac{q}{\sqrt{\pi } }\right)\right)}^{x}{\left(1-erf\left(\frac{h}{\sqrt{\pi } }\right)\right)}^{n-x}$$

The probability of repetition for IC scenario is given as

$${P}_{rep}^{0}=P\left({LCL}_{1}\le X\le {LCL}_{2}\right)+P({UCL}_{2}\le X\le {UCL}_{1})$$

24 $${P}_{rep}^{0}=\sum_{x={UCL}_{2}+1}^{{UCL}_{1}} {\left(erf\left(\frac{q}{\sqrt{\pi } }\right)\right)}^{x}{\left(1-erf\left(\frac{h}{\sqrt{\pi } }\right)\right)}^{n-x} +\sum_{x={LCL}_{1}+1}^{{LCL}_{2}}{\left(erf\left(\frac{q}{\sqrt{\pi } }\right)\right)}^{x}{\left(1-erf\left(\frac{h}{\sqrt{\pi } }\right)\right)}^{n-x}$$

Hence the probability the ongoing process is declared to OOC when in fact it is IC using RSS is given as

25 $${P}_{out}^{0}=\frac{{P}_{out,0}^{0}}{1-{P}_{rep}^{0}}$$

The effectiveness of the implemented CC is evaluated using the ARL. ARL quantifies the average number of subgroups observed before the process is declared as OOC. Hence the IC ARL using RSS is indicated by ${ARL}_{0HND} or {r}_{0HND}$ and calculated as

26 $${ARL}_{0HND}=\frac{1}{{P}_{out}^{0}}$$

The in-control average sample size ${ASS}_{0HND}$ of the proposed chart is given as

27 $${ASS}_{0HND}=\frac{n}{1-{P}_{rep}^{0}}$$

#### Evaluation of the Proposed HND based CC for shifted process

Here, we consider a scenario where the scale parameter of the HND has been shifted due to an external source of variation from ${\alpha }_{0} to{\alpha }_{1}=c{\alpha }_{0}.$ The value of c represents the magnitude of the introduced shift. The probability that an item failing before reaching the specified time ${t}_{0}$ represented by ${P}_{1HND}$ is given as

28 $${P}_{1HND}=P\left(t<{t}_{0}\right)=erf\left(\frac{q}{c\sqrt{\pi } }\right)$$

The probability of the process being in control (IC) when, in reality, it has undergone a switch due to a change in its scale parameter is calculated as

$${P}_{out,1}^{1}= P\left(X<{LCL}_{1}|{P}_{1HND}\right)+P\left(X>{UCL}_{1}|{P}_{1HND}\right)$$

29 $${P}_{out,1}^{1}=\sum_{x=0}^{{LCL}_{1}}\left(\genfrac{}{}{0pt}{}{n}{x}\right) {\left(erf\left(\frac{q}{c\sqrt{\pi } }\right)\right)}^{x}{\left(1-erf\left(\frac{q}{c\sqrt{\pi } }\right)\right)}^{n-x}+\sum_{{d}_{1}={UCL}_{1}+1}^{{LCL}_{1}} \left(\genfrac{}{}{0pt}{}{n}{x}\right) {\left(erf\left(\frac{q}{c\sqrt{\pi } }\right)\right)}^{x}{\left(1-erf\left(\frac{q}{c\sqrt{\pi } }\right)\right)}^{n-x}$$

The probability of repetition for OOC scenario is given as

$${P}_{rep}^{1}=P\left({LCL}_{1}\le X\le {LCL}_{2}\right)+P({UCL}_{2}\le X\le {UCL}_{1})$$

30 $${P}_{rep}^{1}=\sum_{x={UCL}_{2}+1}^{{UCL}_{1}} {\left(eerf\left(\frac{q}{c\sqrt{\pi } }\right)\right)}^{x}{\left(1-erf\left(\frac{q}{c\sqrt{\pi } }\right)\right)}^{n-x} +\sum_{x={LCL}_{1}+1}^{{LCL}_{2}}{\left(erf\left(\frac{q}{c\sqrt{\pi } }\right)\right)}^{x}{\left(1-erf\left(\frac{q}{c\sqrt{\pi } }\right)\right)}^{n-x}$$

Hence the probability the ongoing process is declared to OOC when in fact it is OOC using RSS is given as

31 $${P}_{out}^{1}=\frac{{P}_{out,1}^{1}}{1-{P}_{rep}^{1}}$$

ARL for shifted process under RSS denoted ${ARL}_{1HND}or {r}_{1HND}$ is given as

32 $${ARL}_{1HND}=\frac{1}{{P}_{out}^{1}}$$

The OOC average sample size ${ASS}_{1HND}$ of the proposed chart is given as

33 $${ASS}_{1HND}=\frac{n}{1-{P}_{rep}^{1}}$$

To calculate the ARLs for the proposed control chart, the following algorithm is implemented:

1. Fix the value of IC ARL $say \left({r}_{0HND}\right)$ and $n$
2. Determined the value of $q,{W}_{1} and {W}_{2}$ based on the given sample size $n$ for which ${ARL}_{0HND}$ in Eq. (23) is nearer to ${r}_{0HND}.$
3. Utilize the values of $q, c and n$ obtained in step 2 to calculate the value of ${ARL}_{1HND}$ and ${ASS}_{1HND}$ using Eqs. (29–30) for different values of c.

### Designing of the control chart using Half Exponential Power Distribution

Now, we suppose that the failure time of item follows the HEPD. The probability that an item fails before reaching time ${t}_{0}$ is given by

34 $$P(t < t_{0} ) = \frac{{\gamma \left( {1/\lambda ,t_{0} ^{\lambda } /\lambda \alpha ^{\lambda } } \right)}}{{\Gamma \left( {1/\lambda } \right)}}$$

where ${t}_{0}={q}_{1}{\mu }_{0}$ and ${q}_{1}$ represents the truncated constant for HEPD. Additionally, we substitute the value $\alpha$ in term of $\mu$ using Eq. (11), resulting in the following expression as

35 $$P(t < t_{0} ) = \frac{{\gamma \left( {1/\lambda ,\frac{{q_{1} ^{\lambda } \Gamma \left( {2/\lambda } \right)^{{\uplambda}} }}{{\left( {\Gamma \left( {1/\lambda } \right)} \right)^{{\uplambda}} }}\left( {\frac{{\mu _{0} }}{\mu }} \right)^{\lambda } } \right)}}{{\Gamma \left( {1/\lambda } \right)}}$$

The process is considered to be IC when $\mu ={\mu }_{0}$ (or $\alpha ={\alpha }_{0}and \lambda ={\lambda }_{0}$) then Eq. (34) can be written as

36 $$P_{{0HEPD}} = P_{0} (t < t_{0} ) = \frac{{\gamma \left( {1/\lambda _{0} ,\frac{{q_{1}^{{\lambda _{0} }} \Gamma \left( {1/\lambda _{0} } \right)^{{\lambda _{0} }} }}{{\left( {\Gamma \left( {1/\lambda _{0} } \right)} \right)^{{\lambda _{0} }} }}} \right)}}{{\Gamma \left( {1/\lambda _{0} } \right)}}$$

The inner and outer lower and upper control limits for proposed $np$ charting structure using HEPD under RSS is as follows.

The outer control limits for proposed chart are given as

37 $${LCL}_{1}={\text{Max}}\left[n{P}_{0HEPD}-{W}_{1}\sqrt{n{P}_{0HEPD}\left(1-{P}_{0HEPD}\right)}\right]$$

38 $${UCL}_{1}=n{P}_{0HEPD}+{W}_{1}\sqrt{n{P}_{0HEPD}\left(1-{P}_{0HEPD}\right)}$$

The inner control limits for proposed chart are given as

39 $${LCL}_{2}={\text{Max}}\left[n{P}_{0HEPD}-{W}_{2}\sqrt{n{P}_{0HEPD}\left(1-{P}_{0HEPD}\right)}\right]$$

40 $${UCL}_{2}=n{P}_{0HEPD}+{W}_{2}\sqrt{n{P}_{0HEPD}\left(1-{P}_{0HEPD}\right)}$$

here ${W}_{1} and {W}_{2}$ are the control coefficients with ${W}_{1}$ being greater than ${W}_{2}$. It is important to note the if ${W}_{1}={W}_{2}$ the proposed chart is converted to Naveed et al.^8^ based on HEPD. The working procedure for the presented chart using HEPD is same as we have early discussed for the case of HND. For unknown value of ${P}_{0HEPD}$, the working inner and outer control limits are

41 $${UCL}_{1}=\overline{X }+{W}_{1} \sqrt{\overline{X }\left(1-\overline{X }/n\right)}$$

42 $${LCL}_{1}={\text{Max}}\left[0, \overline{X }-{W}_{1} \sqrt{\overline{X }\left(1-\overline{X }/n\right)}\right]$$

43 $${UCL}_{2}=\overline{X }+{W}_{2} \sqrt{\overline{X }\left(1-\overline{X }/n\right)}$$

44 $${LCL}_{2}={\text{Max}}\left[0, \overline{X }-{W}_{2} \sqrt{\overline{X }\left(1-\overline{X }/n\right)}\right]$$

where $\overline{X }=\frac{\sum X}{n}$ denotes the mean failure time of items before time ${t}_{0}$ in a subgroup over a preliminary sample. The probability of declaring the process as OOC under RSS when it is in fact IC is given as

$${P}_{out,0}^{o}= P\left(X<{LCL}_{1}|{P}_{0HEPD}\right)+P\left(X>{UCL}_{1}|{P}_{0HEPD}\right)$$

45 $${P}_{out,0}^{o}=\sum_{x=0}^{{LCL}_{1}}\left(\genfrac{}{}{0pt}{}{n}{x}\right) {\left({P}_{0HEPD}\right)}^{x}{\left(1-{P}_{0HEPD}\right)}^{n-x}+\sum_{{d}_{1}={UCL}_{1}+1}^{{LCL}_{1}} \left(\genfrac{}{}{0pt}{}{n}{x}\right) {\left({P}_{0HEPD}\right)}^{x}{\left(1-{P}_{0HEPD}\right)}^{n-x}$$

The probability of repetition for IC scenario is given as

$${P}_{rep}^{0}=P\left({LCL}_{1}\le X\le {LCL}_{2}\right)+P({UCL}_{2}\le X\le {UCL}_{1})$$

46 $${P}_{rep}^{0}=\sum_{x={UCL}_{2}+1}^{{UCL}_{1}} {\left({P}_{0HEPD}\right)}^{x}{\left(1-{P}_{0HEPD}\right)}^{n-x} +\sum_{x={LCL}_{1}+1}^{{LCL}_{2}}{\left({P}_{0HEPD}\right)}^{x}{\left(1-{P}_{0HEPD}\right)}^{n-x}$$

Hence the probability the process is declared to OOC when in fact it is IC using RSS is given as

47 $${P}_{out}^{0}=\frac{{P}_{out,0}^{0}}{1-{P}_{rep}^{0}}$$

Hence the IC ARL using HEPD under RSS is indicated by ${ARL}_{0HEPD} or {r}_{0HEPD}$ and calculated as

48 $${ARL}_{0HEPD}=\frac{1}{{P}_{out}^{0}}$$

The in-control average sample size ${ASS}_{0HEPD}$ of the proposed chart based on HEPD is given as

49 $${ASS}_{0HEPD}=\frac{n}{1-{P}_{rep}^{0}}$$

#### Evaluation of the suggested CC using HEPD under RSS when its parameters are shifted

In this section, we examine three different scenarios to evaluate the proposed chart. Each scenario involves a specific type of shift in the parameters of the distribution. These cases are as follow:

##### Shift in scale parameter

In this scenario, we explore the effects of a shift in the scale parameter of the distribution on the performance of the chart, aiming to assess its effectiveness. We consider a situation where the scale parameter, initially represented as ${\alpha }_{0}$ undergoes a transformation to ${\alpha }_{1}$, where ${\alpha }_{1}=e{\alpha }_{0}$ and $e$ represents the magnitude of the introduced shift. The probability of an item failing before the specified time ${t}_{0}$ is denoted by ${P}_{1HEPD}$, which can be calculated as follows:

Rewrite Eq. (35)

$$P\left(t<{t}_{0}\right)=\frac{\gamma \left(1/\lambda ,\frac{{{q}_{1}}^{\lambda }{\Gamma \left(2/\lambda \right)}^{\uplambda }}{{\left(\Gamma \left(1/\lambda \right)\right)}^{\uplambda }}{\left(\frac{{\mu }_{0}}{\mu }\right)}^{\lambda }\right)}{\Gamma \left(1/\lambda \right)}$$

Since, scale level is changed from ${\alpha }_{1}=e{\alpha }_{0}$, as a result the mean value of HEPD is also changed as ${{\mu }_{0}=\mu }_{1}$ by substituting this information in above expression we have

50 $$P_{{1HEPD}} = P(t < t_{0} ) = \frac{{\gamma \left( {1/\lambda _{0} ,q_{1}^{{\lambda _{0} }} \Gamma \left( {2/\lambda _{0} } \right)^{{\lambda _{0} }} /e^{{\lambda _{0} }} \left( {\Gamma \left( {1/\lambda _{0} } \right)} \right)^{{\lambda _{0} }} } \right)}}{{\Gamma \left( {1/\lambda _{0} } \right)}}$$

Now the probability that operation is IC for shifted process due to the change in scale parameter as follows:

$${P}_{out,1}^{1}= P\left(X<{LCL}_{1}|{P}_{1HEPD}\right)+P\left(X>{UCL}_{1}|{P}_{1HEPD}\right)$$

51 $${P}_{out,1}^{1}=\sum_{x=0}^{{LCL}_{1}}\left(\genfrac{}{}{0pt}{}{n}{x}\right) {\left({P}_{1HEPD}\right)}^{x}{\left(1-{P}_{1HEPD}\right)}^{n-x}+\sum_{{d}_{1}={UCL}_{1}+1}^{{LCL}_{1}} \left(\genfrac{}{}{0pt}{}{n}{x}\right) {\left({P}_{1HEPD}\right)}^{x}{\left(1-{P}_{1HEPD}\right)}^{n-x}$$

The probability of repetition for OOC scenario is given as

$${P}_{rep}^{1}=P\left({LCL}_{1}\le X\le {LCL}_{2}\right)+P({UCL}_{2}\le X\le {UCL}_{1})$$

52 $${P}_{rep}^{1}=\sum_{x={UCL}_{2}+1}^{{UCL}_{1}} {\left({P}_{1HEPD}\right)}^{x}{\left(1-{P}_{1HEPD}\right)}^{n-x} +\sum_{x={LCL}_{1}+1}^{{LCL}_{2}}{\left(e{P}_{1HEPD}\right)}^{x}{\left(1-{P}_{1HEPD}\right)}^{n-x}$$

Hence the probability the ongoing process is declared to OOC when in fact it is OOC using RSS is given as

53 $${P}_{out}^{1}=\frac{{P}_{out,1}^{1}}{1-{P}_{rep}^{1}}$$

ARL for shifted process under RSS denoted ${ARL}_{1HEPD}or {r}_{1HEPD}$ is given as

54 $${ARL}_{1HEPD}=\frac{1}{{P}_{out}^{1}}$$

The OOC average sample size ${ASS}_{1HEPD}$ of the proposed chart is given as

55 $${ASS}_{1HEPD}=\frac{n}{1-{P}_{rep}^{1}}$$

Shift in shape parameter.

In this scenario, we consider a situation where the shape parameter of the distribution is shifted. We explore the impact of this adjustment on the probability of an object collapsing before a predefined time period, denoted as ${t}_{0}$. Suppose that the shape parameter, initially represented by ${\lambda }_{0}$ has been modified to ${\lambda }_{1}$, Where ${\lambda }_{1}=r{\lambda }_{0}$. Here, $r$ signifies the extent of the introduced shift. Now, the probability of an object collapsing sooner than the predefined period ${t}_{0}$ designated by ${P}_{2HEPD}$ is calculated as.

Rewrite Eq. (35)

$$P(t < t_{0} ) = \frac{{\gamma \left( {1/\lambda ,\frac{{q_{1} ^{\lambda } \Gamma \left( {2/\lambda } \right)^{{{{\uplambda}}}} }}{{\left( {\Gamma \left( {1/\lambda } \right)} \right)^{{{{\uplambda}}}} }}\left( {\frac{{\mu _{0} }}{\mu }} \right)^{\lambda } } \right)}}{{\Gamma \left( {1/\lambda } \right)}}$$

As shape parameter is shifted from ${\lambda }_{1}=r{\lambda }_{0}$, so its mean level is also changed as ${\mu =\mu }_{1}$ by substituting this information in above expression we have

56 $$P_{{2HEPD}} = P(t < t_{0} ) = \frac{{\gamma \left( {1/\lambda _{1} ,\left( {\frac{{q_{1}^{{\lambda _{1} }} \Gamma \left( {2/\lambda _{1} } \right)^{{\lambda _{1} }} }}{{\left( {\Gamma \left( {1/\lambda _{1} } \right)} \right)^{{\lambda _{1} }} }}} \right)\left( {\frac{{\frac{{\lambda _{0}^{{1/\lambda _{0} }} }}{{\Gamma \left( {1/\lambda _{0} } \right)}}\Gamma \left( {2/\lambda _{0} } \right)}}{{\frac{{\lambda _{1}^{{1/\lambda _{1} }} }}{{\Gamma \left( {1/\lambda _{1} } \right)}}\Gamma \left( {2/\lambda _{1} } \right)}}} \right)^{{\lambda _{1} }} } \right)}}{{\Gamma \left( {1/\lambda _{1} } \right)}}$$

Now the probability that process is IC for shifted process due to the change in shape parameter as follows:

$${P}_{out,1}^{1}= P\left(X<{LCL}_{1}|{P}_{2HEPD}\right)+P\left(X>{UCL}_{1}|{P}_{2HEPD}\right)$$

57 $${P}_{out,1}^{1}=\sum_{x=0}^{{LCL}_{1}}\left(\genfrac{}{}{0pt}{}{n}{x}\right) {\left({P}_{2HEPD}\right)}^{x}{\left(1-{P}_{2HEPD}\right)}^{n-x}+\sum_{{d}_{1}={UCL}_{1}+1}^{{LCL}_{1}} \left(\genfrac{}{}{0pt}{}{n}{x}\right) {\left({P}_{2HEPD}\right)}^{x}{\left(1-{P}_{2HEPD}\right)}^{n-x}$$

The probability of repetition for OOC scenario is given as

$${P}_{rep}^{1}=P\left({LCL}_{1}\le X\le {LCL}_{2}\right)+P({UCL}_{2}\le X\le {UCL}_{1})$$

58 $${P}_{rep}^{1}=\sum_{x={UCL}_{2}+1}^{{UCL}_{1}} {\left({P}_{2HEPD}\right)}^{x}{\left(1-{P}_{2HEPD}\right)}^{n-x} +\sum_{x={LCL}_{1}+1}^{{LCL}_{2}}{\left({P}_{2HEPD}\right)}^{x}{\left(1-{P}_{2HEPD}\right)}^{n-x}$$

Hence the probability the ongoing process is declared to OOC when in fact it is OOC using RSS is given as

59 $${P}_{out}^{1}=\frac{{P}_{out,1}^{1}}{1-{P}_{rep}^{1}}$$

ARL for shifted process under RSS denoted ${ARL}_{2HEPD}or {r}_{2HEPD}$ is given as

60 $${ARL}_{2HEPD}=\frac{1}{{P}_{out}^{1}}$$

The OOC average sample size ${ASS}_{2HEPD}$ of the proposed chart is given as

61 $${ASS}_{2HEPD}=\frac{n}{1-{P}_{rep}^{1}}$$

##### Shift in both parameters

In the last case, we examine a scenario where both the scale and shape parameters of the distribution undergo shifts. This combination of shifts presents a more complex situation, as both the scale and shape parameters of the process are changed simultaneously. We suppose that scale parameter is shifted as ${\alpha }_{1}=q{\alpha }_{0}$ and shape parameter is shifted as ${\lambda }_{1}=w{\lambda }_{0}.$ The probability of an item failing before the predetermined period ${t}_{0}$ designated by ${P}_{3HEPD}$, can now be calculated as:

Rewrite Eq. (35)

$$P\left(t<{t}_{0}\right)=\frac{\gamma \left(1/\lambda ,\left(\frac{{{q}_{1}}^{\lambda }{\Gamma \left(2/\lambda \right)}^{\uplambda }}{{\left(\Gamma \left(1/\lambda \right)\right)}^{\uplambda }}\right){\left(\frac{{\mu }_{0}}{\mu }\right)}^{\lambda }\right)}{\Gamma \left(1/\lambda \right)}$$

As both parameters are shifted as ${\alpha }_{1}=e{\alpha }_{0} and {\lambda }_{1}=r{\lambda }_{0}$, so its mean level is also changed as ${\mu =\mu }_{1}$ by substituting this information in above expression we have

62 $$P_{{3HEPD}} = P(t < t_{0} ) = \frac{{\gamma \left( {1/\lambda _{1} ,\left( {\frac{{q_{1} ^{{\lambda _{1} }} \Gamma \left( {2/\lambda _{1} } \right)^{{\lambda _{1} }} }}{{\left( {\Gamma \left( {1/\lambda _{1} } \right)} \right)^{{\lambda _{1} }} }}} \right)\left( {\frac{{\frac{{\lambda _{0} ^{{1/\lambda _{0} }} }}{{\Gamma \left( {1/\lambda _{0} } \right)}}\Gamma \left( {2/\lambda _{0} } \right)}}{{\frac{{e\lambda _{1} ^{{1/\lambda _{1} }} }}{{\Gamma \left( {1/\lambda _{1} } \right)}}\Gamma \left( {2/\lambda _{1} } \right)}}} \right)^{{\lambda _{1} }} } \right)}}{{\Gamma \left( {1/\lambda _{1} } \right)}}$$

Now the probability for IC when in fact it is OOC as follows.

Now the probability that process is IC for shifted process due to the change in both parameters as follows:

$${P}_{out,1}^{1}= P\left(X<{LCL}_{1}|{P}_{3HEPD}\right)+P\left(X>{UCL}_{1}|{P}_{3HEPD}\right)$$

63 $${P}_{out,1}^{1}=\sum_{x=0}^{{LCL}_{1}}\left(\genfrac{}{}{0pt}{}{n}{x}\right) {\left({P}_{3HEPD}\right)}^{x}{\left(1-{P}_{3HEPD}\right)}^{n-x}+\sum_{{d}_{1}={UCL}_{1}+1}^{{LCL}_{1}} \left(\genfrac{}{}{0pt}{}{n}{x}\right) {\left({P}_{3HEPD}\right)}^{x}{\left(1-{P}_{3HEPD}\right)}^{n-x}$$

The probability of repetition for OOC scenario is given as

$${P}_{rep}^{1}=P\left({LCL}_{1}\le X\le {LCL}_{2}\right)+P({UCL}_{2}\le X\le {UCL}_{1})$$

64 $${P}_{rep}^{1}=\sum_{x={UCL}_{2}+1}^{{UCL}_{1}} {\left({P}_{3HEPD}\right)}^{x}{\left(1-{P}_{3HEPD}\right)}^{n-x} +\sum_{x={LCL}_{1}+1}^{{LCL}_{2}}{\left({P}_{3HEPD}\right)}^{x}{\left(1-{P}_{3HEPD}\right)}^{n-x}$$

Hence the probability the ongoing process is declared to OOC when in fact it is OOC using RSS is given as

65 $${P}_{out}^{1}=\frac{{P}_{out,1}^{1}}{1-{P}_{rep}^{1}}$$

ARL for shifted process under RSS denoted ${ARL}_{3HEPD}or {r}_{3HEPD}$ is given as

66 $${ARL}_{3HEPD}=\frac{1}{{P}_{out}^{1}}$$

The OOC average sample size ${ASS}_{3HEPD}$ of the proposed chart is given as

67 $${ASS}_{3HEPD}=\frac{n}{1-{P}_{rep}^{1}}$$

We will now proceed with the following calculations to create a Table of ARLs for the suggested CC using HEPD for all three cases.

1. Set the values of the ARL $\left({r}_{0HEPD}\right)$, shape parameter, and sample size accordingly.
2. Determine the values of ${q}_{1},{W}_{1} and {W}_{2}$ based on the specified sample size $n$, so that ${ARL}_{0HEPD}$ in Eq. (33) is approaches to ${r}_{0}.$
3. Use the values of ${q}_{1},{W}_{1} and {W}_{2}$ attain in step 2 to calculate the value of ${ARL}_{1HEPD},{ARL}_{2HEPD},{ARL}_{3HEPD}$, for different values of shifted constant.

Record the calculated ARL values in a table, along with the corresponding shifted constants. By following these steps, we can generate the Table of ARLs for the suggested CC using HEPD for all three cases.

## Results discussion

In this section, we discuss the results obtained from two life data distributions. The values of ${ARL}_{1HND},$ ${ARL}_{1HEPD}, {ARL}_{2HEPD}$ and ${ARL}_{3HEPD}$ for various shifts in scale and shape parameters are given in Tables 1, 2, 3, 4, 5, 6, 7, 8, 9 and 10. These Tables display the ARLs of the proposed CC using HND and HEPD for different shifts in shape and scale parameters, as well as sample sizes, when the desired ARLs are set to 300, and 370. By analyzing these tables, we can identify certain patterns in the ARL values of the recommended control chart.

**Figure 3:**
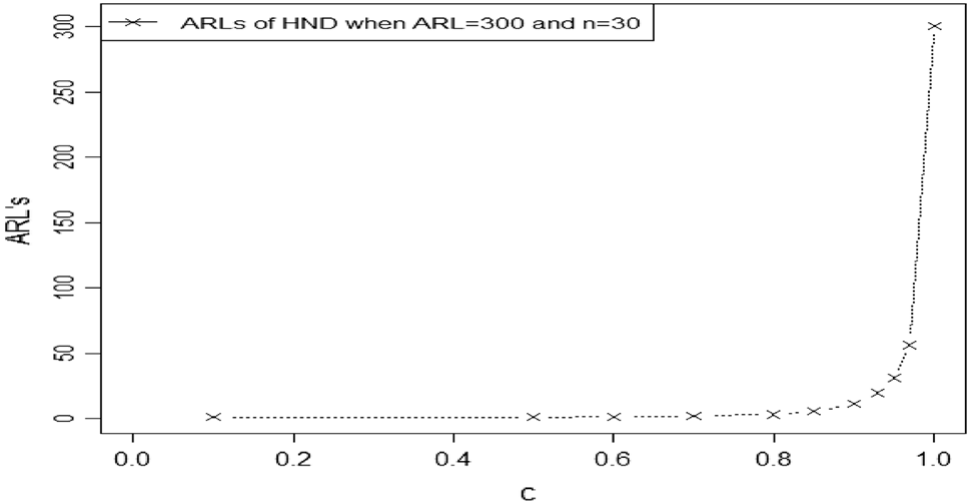
Graph of ARLs for HND using $ARL=300\, {\text{and}}\, n=30$.

**Figure 4:**
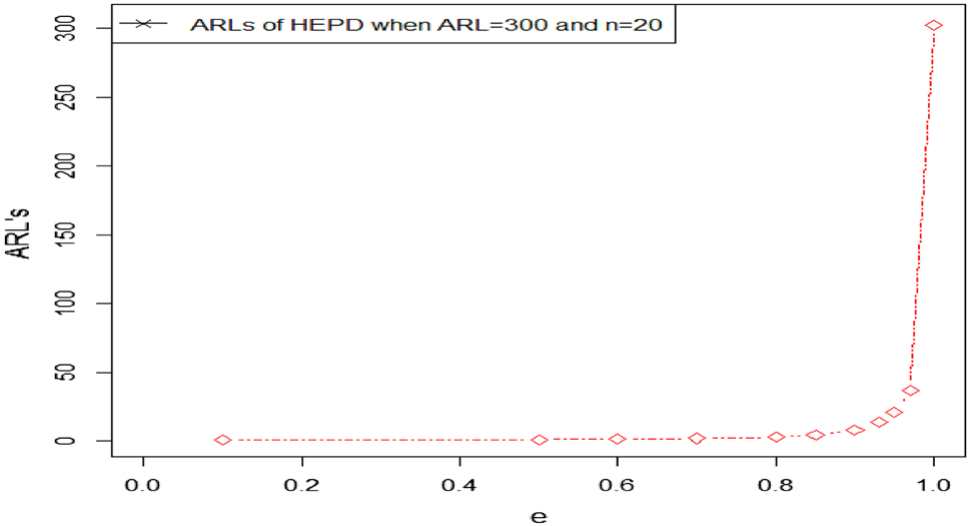
Graph of ARLs for HEPD using $ARL=300,\lambda =4\, {\text{and}}\,n=20$.

1. As we notice that larger shifts in the parameters result in a rapid decrease in the OOC ARL values for both distributions. Let's consider the case of HEPD, where the initial ARL value ${r}_{oHEPD}$ is set to 370, $\lambda$ is 4, shifted constant $e$ is 0.97, and the sample size is 30, the OOC ARL value is 65.45. However, if we decrease $e$ to 0.70 while keeping the other parameters constant, the OOC ARL drops significantly to 1.86. The decreasing trend in ARL for larger shifted values using both HND and HEPD are also shown in Figs. 3 and 4. This demonstrates that larger shifts in the parameter have a notable impact on reducing the value of OOC ARL when the process goes out of control.
2. Furthermore, we observe that increasing the value of shape parameter $\lambda$ leads to a decrease in the value of OOC ARL. For instance, when ${r}_{oHEPD}$ is set to 370, $\lambda$ is 2, shifted constant $e$ is 0.97, and the sample size is 20, the OOC ARL value is 42.38. However, if we increase $\lambda$ to 4 while maintaining the other parameters, the OOC ARL value reduces 38.11. This indicates that a higher value λ leads to a shorter OOC ARL when the process goes out of control

**Table 1:** The ${ARL}_{1HND}\, {\text{and}}\,{ASS}_{1HND}$ values of proposed CC using HND using ${r}_{oHND}=300$.

	$n=20$		$n=30$	
Control limits$\to$	${UCL}_{1}=$ 11.9733 ${LCL}_{1}=$ 6.4012	${UCL}_{2}=$ 11.9713 ${LCL}_{2}=$ 6.4032	${UCL}_{1}=$ 21.8443 ${LCL}_{1}=$ 13.9010	${UCL}_{2}=$ 21.7762 ${LCL}_{2}=$ 13.9690
Control coefficient $\to$	${W}_{1}=$ 1.2500	${W}_{2}=$ 1.2492	${W}_{1}=$ 1.4775	${W}_{2}=$ 1.4522
Truncated constant $\to$	$q=$ 0.7668437		$q=$ 1.045346	

$c$	${ARL}_{1HND}$	${ASS}_{1HND}$	${ARL}_{1HND}$	${ASS}_{1HND}$
1	303.82	24.81	300.48	33.60
0.97	41.01	24.84	56.14	33.88
0.95	23.53	24.87	30.96	34.11
0.93	15.58	24.89	19.43	34.37
0.90	9.61	24.92	11.02	34.80
0.85	5.20	24.89	5.23	35.45
0.80	3.24	24.70	2.93	35.65
0.70	2.23	24.25	1.89	35.07
0.60	1.67	23.51	1.40	33.74
0.50	1.16	21.58	1.05	30.89
0.10	1.02	20.28	1.00	30.03

**Table 2:** The ${ARL}_{1HND} \, {\text{and}}\,{ASS}_{1HND}$ values of proposed CC using HND using ${r}_{oHND}=370$.

	$n=20$		$n=30$	
Control limits$\to$	${UCL}_{1}=$ 12.953 ${LCL}_{1}=7.347$	${UCL}_{2}=$ 12.328 ${LCL}_{2}=7.973$	${UCL}_{1}=$ 19.6406 ${LCL}_{1}=11.565$	${UCL}_{2}=$ 19.2127 ${LCL}_{2}=$ 11.9937
Control coefficient $\to$	${W}_{1}=$ 1.2538	${W}_{2}=$ 0.9740	${W}_{1}=1.4754$	${W}_{2}=1.3191$
Truncated constant $\to$	$q=$ 0.8602		$q=$ 0.7561	

$c$	${ARL}_{1HND}$	${ASS}_{1HND}$	${ARL}_{1HND}$	${ASS}_{1HND}$
1	373.85	24.84	376.80	33.46
0.97	42.83	24.88	71.90	33.61
0.95	23.98	24.91	40.42	33.75
0.93	15.60	24.94	25.80	33.92
0.90	9.43	24.97	14.94	34.22
0.85	5.00	24.95	7.24	34.76
0.80	3.08	24.72	4.04	35.15
0.70	2.11	24.21	2.52	35.12
0.60	1.58	23.38	1.75	34.45
0.50	1.13	21.36	1.14	31.83
0.10	1.01	20.19	1.01	30.20

**Table 3:** The ${ARL}_{1HEPD}\, {\text{and}}\,{ASS}_{1HEPD}$ values of proposed CC using HEPD when its scale parameter is shifted using ${r}_{oHEPD}=300\, {\text{and}}\,n=20$.

	$\lambda =2$		$\lambda =4$	
Control Limits$\to$	${UCL}_{1}=$ 11.9733 ${LCL}_{1}=$ 6.4012	${UCL}_{2}=$ 11.9713 ${LCL}_{2}=$ 6.4032	${UCL}_{1}=11.921$ ${LCL}_{1}=$ 6.453	${UCL}_{2}=11.709$ ${LCL}_{2}=6.6651$
Control Coefficient $\to$	${W}_{1}=$ 1.2500	${W}_{2}=$ 1.2492	${W}_{1}=1.2268$	${W}_{2}=1.1318$
Truncated Constant $\to$	${q}_{1}=0.7668437$		${q}_{1}=0.8570$	

$e$	${ARL}_{1HEPD}$	${ASS}_{1HEPD}$	${ARL}_{1HEPD}$	${ASS}_{1HEPD}$
1	303.82	24.81	302.44	24.81
0.97	41.01	24.84	36.88	24.84
0.95	23.53	24.87	20.80	24.87
0.93	15.58	24.89	13.59	24.90
0.90	9.61	24.92	8.24	24.92
0.85	5.20	24.89	4.37	24.85
0.80	3.24	24.70	2.69	24.52
0.70	2.23	24.25	1.85	23.84
0.60	1.67	23.51	1.41	22.82
0.50	1.16	21.58	1.06	20.75
0.10	1.02	20.28	1.00	20.03

**Table 4:** The ${ARL}_{1HEPD} \, {\text{and}}\, {ASS}_{1HEPD}$ values of proposed CC using HEPD when its scale parameter is shifted using ${r}_{oHEPD}=300 \, {\text{and}}\, n=30$.

	$\lambda =2$		$\lambda =4$	
Control limits$\to$	${UCL}_{1}=$ 21.8443 ${LCL}_{1}=$ 13.9010	${UCL}_{2}=$ 21.7762 ${LCL}_{2}=$ 13.9690	${UCL}_{1}=12.949$ ${LCL}_{1}=$ 5.5664	${UCL}_{2}=12.913$ ${LCL}_{2}=5.6016$
Control coefficient $\to$	${W}_{1}=$ 1.4775	${W}_{2}=$ 1.4522	${W}_{1}=1.4590$	${W}_{2}=1.4451$
Truncated constant $\to$	${q}_{1}==$ 1.045346		${q}_{1}=0.5729$	

$e$	${ARL}_{1HND}$	${ASS}_{1HND}$	${ARL}_{1HEPD}$	${ASS}_{1HEPD}$
1	300.48	33.60	305.65	34.22
0.97	56.14	33.88	53.36	34.35
0.95	30.96	34.11	31.36	34.46
0.93	19.43	34.37	20.96	34.59
0.90	11.02	34.80	12.93	34.79
0.85	5.23	35.45	6.85	35.14
0.80	2.93	35.65	4.09	35.36
0.70	1.89	35.07	2.66	35.27
0.60	1.40	33.74	1.88	34.70
0.50	1.05	30.89	1.20	32.29
0.10	1.00	30.03	1.02	30.34

**Table 5:** The ${ARL}_{1HEPD}\, {\text{and}}\,{ASS}_{1HEPD}$ values of proposed CC using HEPD when its scale parameter is shifted using ${r}_{oHEPD}=370\, {\text{and}}\,n=20$.

	$\lambda =2$		$\lambda =4$	
Control limits$\to$	${UCL}_{1}=$ 12.953 ${LCL}_{1}=7.347$	${UCL}_{2}=$ 12.328 ${LCL}_{2}=7.973$	${UCL}_{1}=$ 11.5427 ${LCL}_{1}=$ 6.8149	${UCL}_{2}=$ 11.4215 ${LCL}_{2}=$ 6.9361
Control coefficient $\to$	${W}_{1}=$ 1.2538	${W}_{2}=$ 0.9740	${W}_{1}=$ 1.0607	${W}_{2}=1.0064$
Truncated constant $\to$	${q}_{1}=0.8602$		${q}_{1}=0.8561$	

$e$	${ARL}_{1HEPD}$	${ASS}_{1HEPD}$	${ARL}_{1HEPD}$	${ASS}_{1HEPD}$
1	373.85	24.84	373.27	24.81
0.97	42.83	24.88	38.11	24.84
0.95	23.98	24.91	21.27	24.87
0.93	15.60	24.94	13.82	24.90
0.90	9.43	24.97	8.35	24.92
0.85	5.00	24.95	4.41	24.85
0.80	3.08	24.72	2.71	24.53
0.70	2.11	24.21	1.86	23.85
0.60	1.58	23.38	1.42	22.83
0.50	1.13	21.36	1.06	20.75
0.10	1.01	20.19	1.00	20.03

**Table 6:** The ${ARL}_{1HEPD}\, {\text{and}}\,{ASS}_{1HEPD}$ values of proposed CC using HEPD when its scale parameter is shifted using ${r}_{oHEPD}=370\, {\text{and}}\,n=30$.

	$\lambda =2$		$\lambda =4$	
Control limits$\to$	${UCL}_{1}=$ 19.6406 ${LCL}_{1}=11.565$	${UCL}_{2}=$ 19.2127 ${LCL}_{2}=$ 11.993	${UCL}_{1}=$ 18.868 ${LCL}_{1}=$ 10.291	${UCL}_{2}=$ 18.321 ${LCL}_{2}=$ 10.838
Control coefficient $\to$	${W}_{1}=1.4754$	${W}_{2}=1.3191$	${W}_{1}=$ 1.5665	${W}_{2}=$ 1.366857
Truncated constant $\to$	${q}_{1}=$ 0.7561		${q}_{1}=$ 0.908059	

$e$	${ARL}_{1HND}$	${ASS}_{1HND}$	${ARL}_{1HEPD}$	${ASS}_{1HEPD}$
1	376.80	33.46	374.85	33.48
0.97	71.90	33.61	65.45	33.66
0.95	40.42	33.75	35.24	33.84
0.93	25.80	33.92	21.70	34.05
0.90	14.94	34.22	12.00	34.42
0.85	7.24	34.76	5.49	35.03
0.80	4.04	35.15	2.97	35.27
0.70	2.52	35.12	1.86	34.69
0.60	1.75	34.45	1.35	33.28
0.50	1.14	31.83	1.03	30.51
0.10	1.01	30.20	1.00	30.00

**Table 7:** The ${ARL}_{2HEPD}\, {\text{and}}\,{ASS}_{2HEPD}$ v values of proposed CC using HEPD when its shape parameter is shifted using ${r}_{oHEPD}=300\, {\text{and}}\,n=20$.

	$\lambda =2$		$\lambda =4$	
Control limits$\to$	${UCL}_{1}=$ 11.9733 ${LCL}_{1}=$ 6.4012	${UCL}_{2}=$ 11.9713 ${LCL}_{2}=$ 6.4032	${UCL}_{1}=11.921$ ${LCL}_{1}=$ 6.453	${UCL}_{2}=11.709$ ${LCL}_{2}=6.6651$
Control coefficient $\to$	${W}_{1}=$ 1.2500	${W}_{2}=$ 1.2492	${W}_{1}=1.2268$	${W}_{2}=1.1318$
Truncated constant $\to$	${q}_{1}=0.7668437$		${q}_{1}=0.8570$	

$r$	${ARL}_{2HEPD}$	${ASS}_{2HEPD}$	${ARL}_{2HEPD}$	${ASS}_{2HEPD}$
1	303.82	24.81	302.44	24.81
1.1	198.94	24.81	129.82	24.81
1.2	142.59	24.81	81.00	24.82
1.3	108.53	24.82	58.21	24.83
1.4	86.19	24.82	45.13	24.84
1.5	70.66	24.82	36.71	24.84
2	34.82	24.85	18.81	24.88
3	16.06	24.89	9.84	24.91
4	10.38	24.91	7.04	24.92
5	7.80	24.92	5.71	24.90

**Table 8:** The ${ARL}_{2HEPD}\, {\text{and}}\,{ASS}_{2HEPD}$ values of proposed CC using HEPD when its shape parameter is shifted using ${r}_{oHEPD}=370\, {\text{and}}\,n=20$.

	$\lambda =2$		$\lambda =4$	
Control limits$\to$	${UCL}_{1}=$ 12.953 ${LCL}_{1}=7.347$	${UCL}_{2}=$ 12.328 ${LCL}_{2}=7.973$	${UCL}_{1}=$ 11.5427 ${LCL}_{1}=$ 6.8149	${UCL}_{2}=$ 11.4215 ${LCL}_{2}=$ 6.9361
Control coefficient $\to$	${W}_{1}=$ 1.2538	${W}_{2}=$ 0.9740	${W}_{1}=$ 1.0607	${W}_{2}=1.0064$
Truncated constant $\to$	${q}_{1}=0.8602$		${q}_{1}=0.8562$	

$r$	${ARL}_{2HEPD}$	${ASS}_{2HEPD}$	${ARL}_{2HEPD}$	${ASS}_{2HEPD}$
1	373.85	24.84	373.27	24.81
1.1	172.14	24.84	142.46	24.81
1.2	109.37	24.85	86.16	24.82
1.3	79.01	24.86	61.03	24.83
1.4	61.23	24.86	46.92	24.84
1.5	49.63	24.87	37.96	24.84
2	24.50	24.91	19.22	24.88
3	11.68	24.96	9.98	24.91
4	7.71	24.98	7.12	24.92
5	5.87	24.97	5.78	24.91

**Table 9:** The ${ARL}_{3HEPD}\, {\text{and}}\,{ASS}_{3HEPD}$ values of proposed CC using HEPD when both parameters are shifted using ${r}_{oHEPD}=300\, {\text{and}}\,n=20$.

		$\lambda =2$		$\lambda =4$	
Control limits$\to$		${UCL}_{1}=$ 11.9733 ${LCL}_{1}=$ 6.4012	${UCL}_{2}=$ 11.9713 ${LCL}_{2}=$ 6.4032	${UCL}_{1}=11.921$ ${LCL}_{1}=$ 6.453	${UCL}_{2}=11.709$ ${LCL}_{2}=6.6651$
Control coefficient $\to$		${W}_{1}=$ 1.2500	${W}_{2}=$ 1.2492	${W}_{1}=1.2268$	${W}_{2}=1.1318$
Truncated constant $\to$		${q}_{1}=0.7668437$		${q}_{1}=0.85702$	

$e$	$r$	${ARL}_{3HEPD}$	${ASS}_{3HEPD}$	${ARL}_{3HEPD}$	${ASS}_{3HEPD}$
1	1	303.82	24.81	302.44	24.81
1.1	0.97	36.72	24.84	29.93	24.85
1.2	0.95	19.86	24.87	15.69	24.89
1.3	0.93	12.55	24.90	9.81	24.91
1.4	0.90	7.52	24.92	5.92	24.91
1.5	0.85	4.02	24.82	3.23	24.69
2	0.80	2.20	24.22	1.84	23.81
3	0.70	1.16	21.59	1.09	21.06
4	0.60	1.00	20.07	1.00	20.02
5	0.50	1.00	20.00	1.00	20.00

**Table 10:** The ${ARL}_{3HEPD}\, {\text{and}}\,{ASS}_{3HEPD}$ values of proposed CC using HEPD when both parameters are shifted using ${r}_{oHEPD}=370\, {\text{and}}\,n=20$.

		$\lambda =2$		$\lambda =4$	
Control limits$\to$		${UCL}_{1}=$ 12.953 ${LCL}_{1}=7.347$	${UCL}_{2}=$ 12.328 ${LCL}_{2}=7.973$	${UCL}_{1}=$ 11.5427 ${LCL}_{1}=$ 6.8149	${UCL}_{2}=$ 11.4215 ${LCL}_{2}=$ 6.9361
Control coefficient $\to$		${W}_{1}=$ 1.2538	${W}_{2}=$ 0.9740	${W}_{1}=$ 1.0607	${W}_{2}=1.0064$
Truncated constant $\to$		${q}_{1}=0.8602$		${q}_{1}=0.8561$	

$e$	$r$	${ARL}_{3HEPD}$	${ASS}_{3HEPD}$	${ARL}_{3HEPD}$	${ASS}_{3HEPD}$
1	1	373.8	24.8	373.27	24.81
1.1	0.97	35.3	24.9	30.80	24.85
1.2	0.95	18.2	24.9	15.99	24.89
1.3	0.93	11.2	25.0	9.96	24.91
1.4	0.90	6.6	25.0	5.98	24.91
1.5	0.85	3.5	24.8	3.26	24.70
2	0.80	1.9	23.9	1.85	23.82
3	0.70	1.1	20.9	1.10	21.07
4	0.60	1.0	20.0	1.00	20.02
5	0.50	1.0	20.0	1.00	20.00

## Advantages of recommended control chart

In this section, we conducted several comparisons between the proposed control charts (CC) and other existing charts, including the Shewhart type chart proposed by Naveed et al.^8^ and the chart proposed by Aslam and Jun^1^. Additionally, we compared the suggested CCs with the Exponential Distribution (ED) CC under repetitive sampling. The evaluation of these control charts is based on their ARL values, which are presented in Table 11.

**Table 11:** Comparison of ARLs when ${r}_{o}=370\, {\text{and}}\,n=20$.

	Proposed HEPD when scale parameter is shifted using $\lambda =$ 4	Proposed chart based on HND	Exponential distribution based CC using RSS	Existing chart proposed by Naveed et al.^8^ based on HEPD	Existing chart proposed by Naveed et al.^8^ based on HND	Existing chart proposed by Aslam and Jun^1^
Control coefficient $\to$	${W}_{1}=$ 1.0607 ${W}_{2}=1.0064$	${W}_{1}=$ 1.2538 ${W}_{2}=$ 0.974	${W}_{1}=1.466$ ${W}_{2}=1.469$	$k=$ 3.123	$k=$ 3.184	$k=3.214$
Truncated constant $\to$	${q}_{1}=0.8561$	$q=0.8602$	$h=0.73433$	$h=0.3728$	$h=$ 0.3192	$h=0.2244$

Shifts	$ARL$	$ARL$	$ARL$	$ARL$	$ARL$	$ARL$
1	373.27	373.85	371.05	370.26	370.16	370.571
0.97	38.11	42.83	90.12	292.24	293.62	300.077
0.95	21.27	23.98	52.22	248.94	250.99	260.157
0.93	13.82	15.60	33.93	211.59	214.12	225.166
0.90	8.35	9.43	20.02	165.13	168.09	180.721
0.85	4.41	5.00	9.95	108.03	111.15	124.189
0.80	2.71	3.08	5.64	69.69	72.57	84.412
0.70	1.86	2.11	3.51	27.85	29.84	37.758
0.60	1.42	1.58	2.37	10.65	11.81	16.256
0.50	1.06	1.13	1.38	4.05	4.65	6.854
0.10	1.00	1.01	1.07	1.00	1.00	1

**Figure 5:**
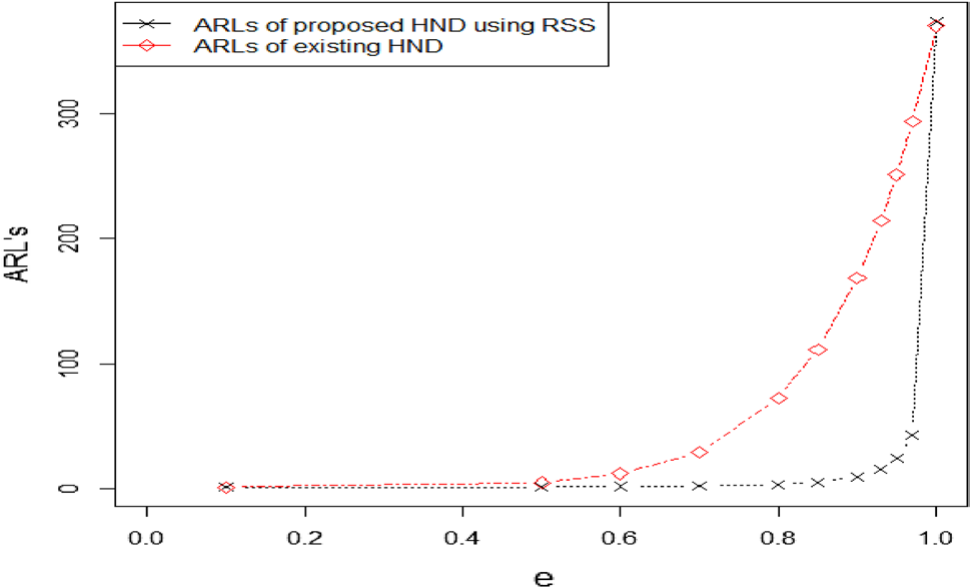
Graph of ARLs for proposed HND verse^8^ using $ARL=370\, {\text{and}}\,n=20$.

**Figure 6:**
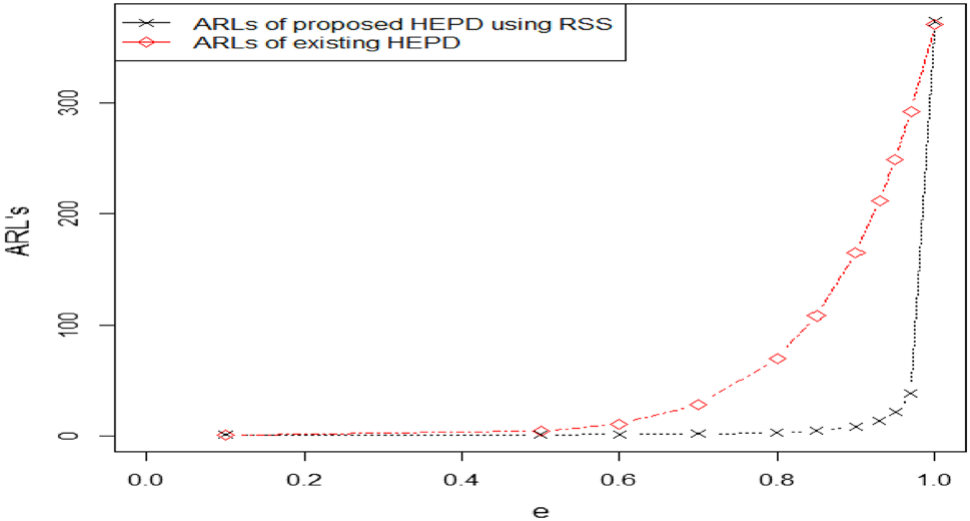
Graph of ARLs for proposed HEPD verse^8^ using $ARL=370 , \lambda =4\, {\text{and}}\,n=20$.

Upon analyzing Table 11, it is evident that both RSS based control charts outperformed the Naveed et al.^8^ chart. For example, when considering the proposed control chart based on the HND with the following parameters: ${r}_{0HND}=370, n=20, q= 0.8602,{W}_{1}=1.2538$ ${W}_{2}=0.974$ and shifted constant $c=0.95$ the value of OOC ARL for proposed control chart is 23.98. In comparison, the OOC ARL value for the chart proposed by Naveed et al.^8^ was 250.99. The comparison of these two charts is also shown in Fig. 5. Similarly, for the proposed chart based on HEPD with parameters ${r}_{0HEPD}=370, n=20, q= 0.8561334,{W}_{1}=1.0607,{W}_{2}=$ 1.0064 and shift in scale parameter e $=0.93$ the value of ARL for proposed control chart is 13.82 and for Naveed et al.^8^ it was 211.59 using HEPD. The comparison of these two charts is also shown in Fig. 6.

Furthermore, we observed that the proposed HEPD-based CC performed better than the proposed HND-based chart, and both of these proposed charts demonstrated superior performance compared to the ED based chart under RSS. For example, when ${r}_{0}=370, n=20, q= 0.8561334,{W}_{1}=1.0607,{W}_{2}=$ 1.0064 and the shift in scale parameter $e=0.95$ the value of ARL in the proposed control chart using HEPD is 21.27 and for HND the value of ARL is 23.98 using $n=20, q= 0.8602,{W}_{1}=$ 1.2538 $,{W}_{2}=$ 0.974 and shifted constant c $=0.95.$ and in the cases of competitor exponential distribution based chart it was 52.22.

Additionally, we observed that the proposed control charts exhibited smaller ARL values compared to the charts proposed by Aslam and Jun^1^. For example, when utilizing the proposed control chart based on HEPD with parameters when ${r}_{0}=370, n=20, q= 0.8561334,{W}_{1}=1.0607,{W}_{2}=$ 1.0064 and the shift in scale parameter e $=0.93$ the value of ARL for the proposed control chart using HEPD is 13.82 and for HND the value of ARL is 15.60 using $n=20, q= 0.8602,{W}_{1}=$ 1.2538 $,{W}_{2}=$ 0.974 and shifted constant c $=0.93.$ and for the cases of Aslam and Jun^1^ it was 225.16. Overall, these comparisons demonstrate the superior performance of the proposed control charts in detecting variations and maintaining process control compared to the alternative approaches proposed by Aslam and Jun^1^ and Naveed et al.^8^, as well as the Exponential Distribution based control chart under repetitive sampling.

## Simulation study

**Figure 7:**
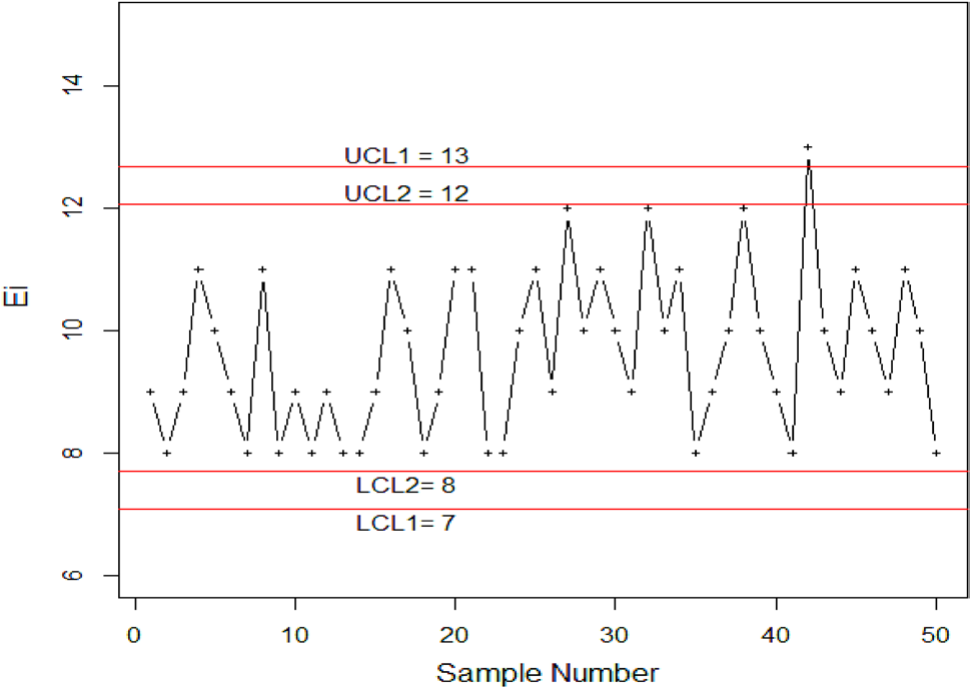
Simulated data for proposed chart.

**Figure 8:**
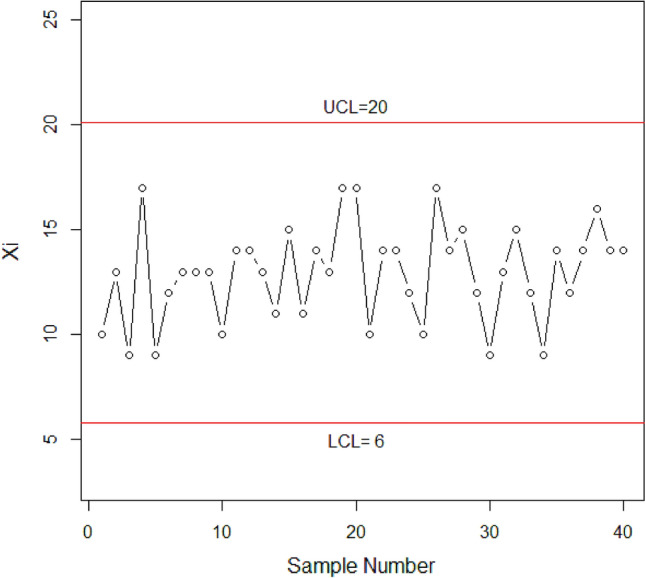
Simulated data for the chart proposed by Naveed et al.^8^.

In this section, we demonstrate the effectiveness of the proposed idea through the use of simulation data. We assume that the process is initially operating under stable conditions, and we generate 25 observations of subgroup size 20 from the HEPD using the RSS. The in-control scale parameter is set to $\alpha =1$, and the shape parameter is set to $\lambda =2$. Next, we introduce a disruption to the stable process by altering its scale parameter due to external factors. The new scale parameter, denoted as ${\alpha }_{1}$, is defined as ${\alpha }_{1}=e{\alpha }_{0}$, where ${\alpha }_{0}$ is the initial scale parameter and $e=0.95$ is the shifted constant. We generate another set of 25 observations of subgroup size 20 from the HEPD using RSS, this time with ${\alpha }_{1}=0.80$ and $\lambda =2$, representing the shifted process. We suppose that ${ARL}_{0HEP}=370$. The truncated time ${t}_{0}={(q}_{1}*\mu )$, calculated as using parameter $\alpha =1,\lambda =2$ and ${r}_{0}=370$, gives the value of ${q}_{1}$ from Table 5 as ${q}_{1}=0.8602$, and value of $\mu$ using Eq. (11) against parameter $\alpha =1,\lambda =2$ calculated as $\mu =0.7978$ and truncation time ${t}_{0}=0.8602*0.7978=0.6863.$ The number of items which are failing before the time 0.6863 in each subgroup is designated by X and reported in Table 12. Using these values, we calculate the control limits from Eqs. (19–22) with ${W}_{1}=$1.2538, ${W}_{2}=$0.9740 as ${LCL}_{1}=7, {UCL}_{1}=12 ,{LCL}_{2}=8, {UCL}_{2}=13.$ To visualize the data set of failed items, we plot the values in Fig. 7. The plot shows that the process is disrupted at the 41-th item, which corresponds to the 16-th observation after the shift in the process. This result is consistent with the value reported in Table 5. We also plot the data set of competitor chart proposed by Naveed et al.^8^ which shows the in-control process in Fig. 8. Therefore, it is evident that the suggested control chart efficiently detects the early shift in the process. Overall, this analysis demonstrates the capability of the proposed control chart in identifying process disruptions and detecting shifts in a timely manner using simulation data.

**Table 12:** Simulated data.

$X$	$X$	$X$	$X$	$X$
9	8	9	11	10
9	8	11	8	9
8	9	8	8	9
11	10	8	9	11
11	8	8	10	11
9	12	10	11	10
9	12	10	11	8
9	10	12	10	9
8	13	10	9	11
10	9	11	10	8

## Real life examples

**Figure 9:**
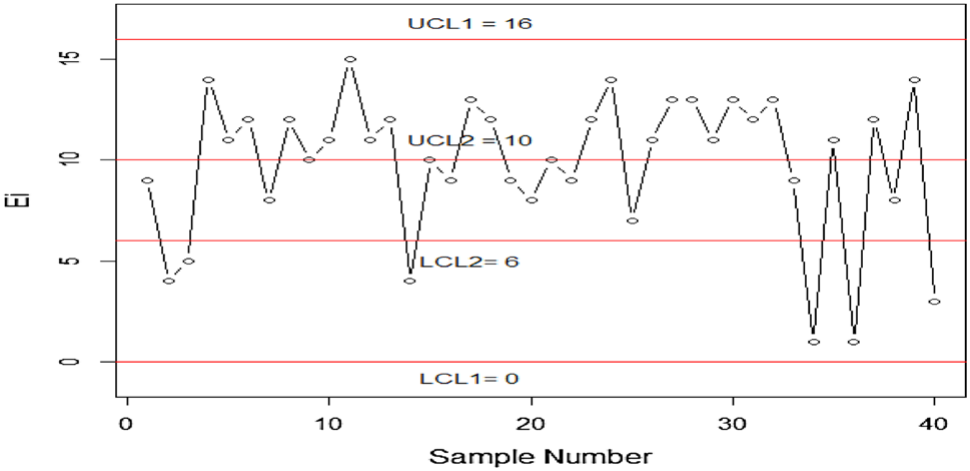
Graph of proposed CC using HEPD with real life data application.

The proposed control chart is applied and evaluated using real-life data obtained from a study conducted by Gui^35^. The dataset consists of plasma ferritin cluster measurements of 202 athletes collected at the Australian Institute of Sport. This dataset has been previously analyzed by multiple researchers, including Naveed et al.^8^, Elal-Olivero et al.^27^, Azzalini and Valle^38^ and Cook and Weisberg^39^. The data follows a HEPD with a mean of 76.88 plasma ferritin and a standard deviation of 47.50 plasma ferritin. The known scale parameter, $\alpha$, is 97.1311, and the shape parameter, $\lambda$, is 2.5109. Assuming ${r}_{0HEPH}=300,n=30$ and ${q}_{1}=0.49785$, we can obtain the value of ${P}_{0}$ using Eq. (36) as ${P}_{0}=0.2913.$ The The inner and outer control limits using Eqs. (37–40) are ${LCL}_{1}=0, {UCL}_{1}=16, {LCL}_{2}=6, {UCL}_{2}=10$ respectively, when ${W}_{1}=3.185, {W}_{2}=0.844$. The truncated time ${t}_{0}={q}_{1}*{\mu }_{0}=0.49785*76.88=38.27$ plasma ferritin. The following steps describe how the chart works:

Stage 1: A sample of 30 items is randomly selected from the HEPD distribution with a scale parameter $\alpha =97.1311$ and a shape parameter $\lambda =2.5109$. These items are then placed on a truncated time ${t}_{0}=38.27$ plasma ferritin. The number of failed items (X) is counted during the test, and the results are plotted in Fig. 9.

Step 2: Declare the running process is under control if $6\le X\le 10$.

Step 3: The process is considered to be out if control if $X>16 or X<0$.

Step 4: If the value of $X$ lies in repetitive mode i.e. $0\le X\le 6 or 10\le X\le 16$ then we take next values of $X$ until we reach a decision of in-control or out of control process.

## Concluding remarks

In this research paper, we introduced two attribute control charts specifically designed to detect early changes in process parameters. These charts are tailored for the HND and HEPD under the TTLT using repetitive sampling. We evaluated the performance of these charts by comparing them to existing competitor charts based on the ARL metric. The results of our study demonstrated that the proposed control charts exhibit superior performance in identifying minor process changes, as evidenced by their lower ARL values. This conclusion is supported by both simulation-based experiments and real-life datasets. The promising results obtained from these evaluations highlight the effectiveness and relevance of our proposed control charting approach. In the context of these two proposed distributions, our analysis highlighted that the CC stemming from the HEPD exhibited superior performance in detecting smaller shifts within process parameter as compared to half normal distribution-based CC. Furthermore, we believe that this research opens up avenues for future investigations. In conclusion, our study introduces a valuable contribution to the field of control charts by presenting two attribute control charts specifically designed for the HND and HEPD under TTLT using repetitive sampling. The superior performance of these charts in detecting early process changes, as supported by simulations and real-life data, confirms their potential as effective tools for quality control. Investigating the control chart employing the cost model is a potential avenue for future research. Furthermore, exploring the control chart utilizing multiple dependent state repetitive sampling is another promising direction for future studies.

## Data Availability

All data is given in the paper.
